# Calcium in a supernova remnant as a fingerprint of a sub-Chandrasekhar-mass explosion

**DOI:** 10.1038/s41550-025-02589-5

**Published:** 2025-07-02

**Authors:** Priyam Das, Ivo R. Seitenzahl, Ashley J. Ruiter, Friedrich K. Röpke, Rüdiger Pakmor, Frédéric P. A. Vogt, Christine E. Collins, Parviz Ghavamian, Stuart A. Sim, Brian J. Williams, Stefan Taubenberger, J. Martin Laming, Janette Suherli, Ralph Sutherland, Nicolás Rodríguez-Segovia

**Affiliations:** 1 School of Science, University of New South Wales, Australian Defence Force Academy, Canberra, Australian Capital Territory Australia; 2 Heidelberger Institut für Theoretische Studien, Heidelberg, Germany; 3 OzGrav: The ARC Centre of Excellence for Gravitational Wave Discovery, Hawthorn, Victoria Australia; 4 ARC Centre of Excellence for All-Sky Astrophysics in 3 Dimensions, Canberra, Australian Capital Territory Australia; 5 Zentrum für Astronomie der Universität Heidelberg, Institut für Theoretische Astrophysik, Heidelberg, Germany; 6 Zentrum für Astronomie der Universität Heidelberg, Astronomisches Recheninstitut, Heidelberg, Germany; 7 Max-Planck-Institut für Astrophysik, Garching, Germany; 8 Federal Office of Meteorology and Climatology – MeteoSwiss, Payerne, Switzerland; 9 School of Physics, Trinity College Dublin, The University of Dublin, Dublin, Ireland; 10 GSI Helmholtzzentrum für Schwerionenforschung, Darmstadt, Germany; 11 Department of Physics Astronomy and Geosciences, Towson University, Towson, MD USA; 12 School of Mathematics and Physics, Queen’s University Belfast, Belfast, UK; 13 X-ray Astrophysics Laboratory, NASA/GSFC, Greenbelt, MD USA; 14 Max-Planck-Institute for Astrophysics, Garching, Germany; 15 TUM Department of Physics, Technical University Munich, Garching, Germany; 16 Space Science Division, Code 7684, Naval Research Laboratory, Washington, DC USA; 17 Department of Physics and Astronomy, University of Manitoba, Winnipeg, Manitoba Canada; 18 Research School of Astronomy and Astrophysics, Australian National University, Weston Creek, Australian Capital Territory Australia

**Keywords:** Stellar evolution, Time-domain astronomy

## Abstract

Type Ia supernovae play a fundamental role as cosmological probes of dark energy and produce more than half of the iron in our Galaxy. Despite their central importance, a comprehensive understanding of their progenitor systems and triggering mechanism is still a long-standing fundamental problem. Here we present high-resolution integral field spectroscopic observations of the young supernova remnant SNR 0509-67.5 in the Large Magellanic Cloud. We uncover a double-shell morphology of highly ionized calcium [Ca XV] and a single shell of sulphur [S XII], observed in the reverse shocked ejecta. Our analysis reveals that the outer calcium shell originates from the helium detonation at the base of the outer envelope, while the inner shell is associated with the carbon–oxygen core detonation. This morphological distribution of intermediate-mass elements agrees qualitatively with the predicted signature of the double detonation of a sub-Chandrasekhar-mass white dwarf from a hydrodynamical explosion simulation. Our observations reveal two distinct, spatially separated peaks in surface brightness of [Ca XV] from the supernova remnant phase, providing substantial evidence that sub-Chandrasekhar-mass explosions through the double-detonation mechanism could occur in nature. They also highlight the importance of remnant tomography in understanding explosion mechanisms from the remnant phase.

## Abstract (web summary)

Observations of the young supernova remnant SNR 0509-67.5 in the Large Magellanic Cloud reveal concentric shells of ionized calcium and sulfur that resemble hydrodynamical simulations of the double detonation of a sub-Chandrasekhar-mass white dwarf.

## Main

The question of how a thermonuclear explosion initiates in an inert object such as a white dwarf (WD) star is an essential and long-standing problem in stellar astrophysics^1^. In a WD consisting of carbon and oxygen and approaching the Chandrasekhar mass, the increasing central density inevitably triggers nuclear burning. The almost constant explosion mass that the Chandrasekhar-mass explosion model provides was a popular explanation for the homogeneity initially attributed to type Ia supernovae (SNe Ia)^2^. However, mounting observational data challenge the notion of type Ia supernova (SN Ia homogeneity^3,4^, even for the spectroscopically normal ones (that is, Wang et al.^5,6^, who identified a high-velocity population among normal SNe Ia), and a fixed mass even seems problematic for reproducing the width–luminosity relation^7^, which is vital for calibrating type Ia supernovae as cosmological distance indicators. The width–luminosity relation is more naturally explained by a variable WD mass below the Chandrasekhar-mass limit as the primary parameter^8,9^. Moreover, the ability to grow WDs to the Chandrasekhar mass restricts the parameters of the progenitor binary system to a narrow range, so that the observed rate of type Ia supernovae is hard to reconcile with the expected number of systems consistent with the Chandrasekhar-mass explosion scenario^10,11^. This calls for alternative scenarios involving explosions of carbon–oxygen WD stars well below the Chandrasekhar-mass limit and raises the fundamental problem of how to ignite a thermonuclear explosion in an inert sub-Chandrasekhar-mass WD.

A head-on collision of two WDs may seem promising as a pathway for producing sub-Chandrasekhar-mass exploding WDs^12^, but this scenario is not favoured because the predicted occurrence rates are too low^13^. The currently most promising scenario for exploding sub-Chandrasekhar-mass WDs is double detonation, where a carbon–oxygen WD collects helium-rich material from a non-degenerate or degenerate companion (from a helium star or a helium-rich WD, or from the pre-existing thin helium layer on top of a carbon–oxygen WD in merger events^14–16^). In this helium layer, a detonation is triggered either by compressional heating when the helium layer becomes sufficiently massive or owing to dynamical instabilities^17–20^. This first detonation propagates through the thin helium layer and drives a shock wave into the carbon–oxygen core, where it focusses spherically into a small volume. The compression and heating of the carbon–oxygen material in this region initiates a secondary detonation in the core material and successfully explodes the sub-Chandrasekhar-mass WD^21^. Although numerous simulations indicate that the double-detonation mechanism is feasible, so far they have failed to resolve the spatial length scales on which the primary helium detonation ignites^22,23^. While unable to demonstrate the ignition of the required detonations, these simulations do provide us with critical information about the structure, morphology and early time spectra of a double-detonation type Ia supernova if the ignitions of both detonations are successful.

In terms of double-detonation nucleosynthesis, the detonations in the carbon–oxygen core and the outer helium-rich layer result in qualitatively different yield products. This should not come as a surprise, since both the type of fuel (carbon–oxygen versus helium) and the densities (higher density in the core and lower density in the outer layer) differ substantially, by about two orders of magnitude. In the core, the density of the fuel is the key parameter that determines the outcome of the explosive nuclear burning. For densities greater than ~7 × 10^6^ g cm^−3^, the burning is nearly complete, and iron-group elements (IGEs), especially the radioactive ^56^Ni nucleus, dominate the nucleosynthetic yields. At the ‘intermediate’ densities further off-centre in the core, the nuclear fusion time scale becomes increasingly longer and the rapid expansion of the WD leads to a freeze-out of the nuclear reactions before burning to IGEs is completed. As a result, the synthesis of intermediate-mass elements (IMEs) dominates these regions, with heavier IMEs such as calcium relatively more abundant further inside and lighter IMEs such as silicon or sulfur becoming relatively more abundant as the fuel density further decreases outwards. Eventually, the density becomes too low (~3 × 10^6^ g cm^−3^) for oxygen to burn and only carbon continues to burn to light IMEs such as oxygen, neon and magnesium. A recent review^24^ shows a schematic of this well-known layered structure.

**Extended Data Fig. 1:**
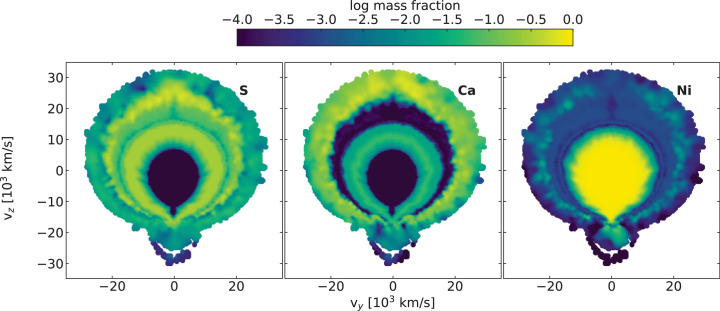
Slices of the M10_03 explosion model. Slices through the y-z plane of the M10_03 double- detonation hydrodynamical explosion model? ? show the distribution of sulfur (left), calcium (middle), and nickel (right) in velocity space. The model starts with a C-O core of 1 M_⊙_ and a helium layer of 0.03 M_⊙_ to create the double detonation simulation.

At even lower densities, the fuel composition rapidly changes where the helium layer (sometimes referred to as a shell) begins. Importantly, owing to its lower Coulomb barrier, helium (^4^He) is more reactive, and helium detonations are possible down to much lower densities^25^. Similar to the carbon–oxygen core, ‘helium-shell’ detonations produce a radially layered progression in the atomic weight of the burning products, with the heavier elements such as chromium, iron or nickel preferentially synthesized at the inner, denser parts of the helium shell; lighter elements such as unburned helium, carbon or oxygen are found at the outer, less dense parts of the helium shell, and intermediate-mass elements such as silicon or sulfur in between^26^ (Extended Data Fig. 1). For optimal agreement with observations (in particular, the colours in synthetic lightcurves), it is important that the density at the base of the helium shell is not too large (less than ~10^6^ g cm^−3^), such that the production of IGEs in the He shell is limited and intermediate-mass elements such as calcium are the most abundant nucleosynthesis products at the base of the helium shell^20,27^.

Therefore, taking the nucleosynthetic signatures of the CO core and the He shell together, double-detonation models predict calcium to be concentrated in two separate layers: an inner layer from the core region, corresponding to the incomplete burning of the CO detonation (at fuel densities around a few 10^6^ g cm^−3^), and an outer layer at higher velocity in the expanding explosion ejecta, corresponding to the base of the He shell (fuel densities around a few 10^5^ g cm^−3^). Explosion models, including the M10_03 model by Collins et al.^28^ (Extended Data Fig. 1), predict such a double shell morphology of Ca, with intermediate-mass elements lighter than Ca, such as S or Si, located in between the two shells.

While numerical simulations alone cannot confirm that the double-detonation mechanism occurs in nature, a confirmed observation of the tell-tale two-shell structure would supply direct evidence for its operation in type Ia supernovae. One indirect observational signature supporting the double-detonation mechanism includes the detection of intermediate-mass elements at appropriately high velocities, and plausible evidence for double-detonation events has been previously discussed in the context of high velocity features (HVFs)^27,29,30^. In particular, detached HVFs of Ca II suggest that distinct line-forming regions are present (as both a photospheric and a high-velocity component), as qualitatively suggested by the nucleosynthesis pattern expected from a double detonation. The HVFs of Ca II and Si II were studied in a sample of 445 SNe at epochs up to 5 days past maximum brightness^31^. HVFs of Ca II were found in almost two-thirds of the sample^31^, but interestingly such features were absent from the 91bg-like (faint) sub-class of SNe Ia. Additionally, investigation of high-velocity features (HVFs) in type Ia supernovae revealed that Si II and Ca II HVFs are more prevalent in overluminous, slowly declining SNe Ia (for example, 91T-like events), correlating with lower Δm_15_(B) (decline within 15 d from peak in the B band), while such features are absent in high-velocity (*v*_Si_ ≥ 12,000 km s^−1^) SNe^32^. An inverse correlation between [O I] (*λ*7,773) HVFs and Si/Ca II HVFs in normal SNe was observed, linking stronger oxygen absorption to less complete burning and lower luminosities^33^. [Ca II] has also been observed at later times in the spectra of SN 2019yvq^34,35^, SN 2018byg^36^ and SN 2016hnk^37^. To date, supernova SN2018byg is widely acknowledged as one of the most compelling cases linking the double-detonation mechanism to a SNe Ia explosion and is best explained by a model that incorporates a rather massive helium layer^36^. However, quantitative interpretation of HVFs depends on spectral modelling, and a range of possible alternative interpretations for these spectroscopic features have been presented. For example, while it has been suggested that the HVFs may be connected to a surface layer of helium, it has been argued that abundance enhancements alone cannot explain the full range of such features^38^ and that density enhancement in the outer ejecta might originate, for example, from interaction with circumstellar material in addition to (or instead of) properties of the explosion mechanism^39^. Despite advances in observations, modelling inconsistencies remain, particularly for 91T-like SNe Ia. Current one-dimensional double-detonation models^29^ struggle to reproduce their normal-velocity Si II and high luminosity, instead predicting low-luminosity, high-velocity events. Recent non-local thermodynamic equilibrium simulations show promise in better matching observations^40^, highlighting the need for more multi-dimensional and detailed radiative transfer studies.

Owing to the small angular size at early times, the unique double-shell Ca morphology ‘fingerprint’ structure remains spatially unresolved at the epochs around peak luminosity (15–20 days after explosion), which is why any inference of a double-shell ejecta structure from observations at such phases depends on interpreting spectral features (as outlined above). However, this changes with time as the supernova continually expands. Here, we present new evidence—a spatially resolved ‘photographic snapshot’ of a double calcium shell—showing that SNe Ia can explode via the double-detonation mechanism. The evidence is based on ~29 h 15 min deep Multi Unit Spectroscopic Explorer (MUSE) integral field observations of the reverse shocked ejecta of the supernova remnant SNR 0509-67.5 (hereafter SNR 0509). The details of the nights over which the data were collected are given in Table 1.

**Table 1:** Observation log

Number of observation	Date of observation	Exposure time (s)	Airmass	DIMM seeing at start (arcsec)
1	8 February 2021	2,700	1.364	0.68
2	8 February 2021	2,700	1.389	0.49
3	7 February 2021	2,700	1.367	0.51
4	7 February 2021	2,700	1.397	0.61
5	6 February 2021	2,700	1.384	0.37
6	5 February 2021	2,700	1.396	0.67
7	16 January 2021	2,700	1.402	0.52
8	12 January 2021	2,700	1.381	0.72
9	11 January 2021	2,700	1.395	0.60
10	10 January 2021	2,700	1.396	0.66
11	17 December 2020	2,700	1.365	0.53
12	17 December 2020	2,700	1.375	0.44
13	16 December 2020	2,700	1.375	0.56
14	15 December 2020	2,700	1.398	0.53
15	15 December 2020	2,700	1.375	0.45
16	14 December 2020	2,700	1.406	0.35
17	14 December 2020	2,700	1.370	0.61
18	13 December 2020	2,700	1.370	0.66
19	13 December 2020	2,700	1.394	0.32
20	13 December 2020	2,700	1.411	0.49
21	13 December 2020	2,700	1.365	0.52
22	12 December 2020	2,700	1.365	0.53
23	12 December 2020	2,700	1.495	0.46
24	12 December 2020	2,700	1.395	0.60
25	12 December 2020	2,700	1.369	0.48
26	10 December 2020	2,700	1.391	0.67
27	22 November 2020	2,700	1.364	0.42
28	20 November 2020	2,700	1.367	0.60
29	20 November 2020	2,700	1.369	0.47
30	15 November 2020	2,700	1.404	0.48
31	14 November 2020	2,700	1.441	0.47
32	14 November 2020	2,700	1.382	0.51
33	13 November 2020	2,700	1.419	0.50
34	13 November 2020	2,700	1.371	0.61
35	12 November 2020	2,700	1.385	0.51
36	12 November 2020	2,700	1.363	0.47
37	16 February 2020	2,700	1.456	0.39
38	23 December 2019	2,700	1.396	0.37
39	26 November 2019	2,700	1.371	0.36

The nights when the target was observed and information about the quality of the observation. DIMM, Differential Image Motion Monitor.

## Results

**Fig. 1:**
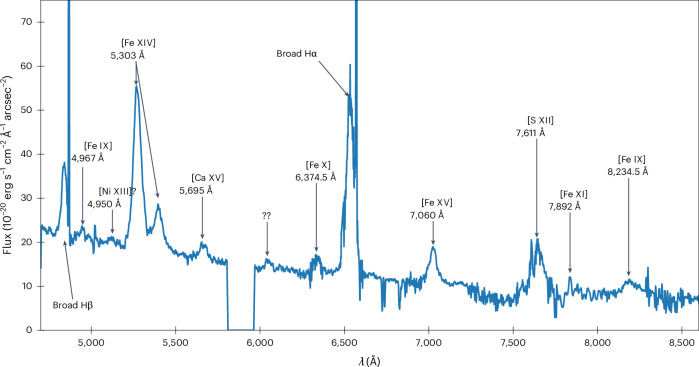
Spectral features of SNR 0509, showing coronal lines and Balmer emission. A spectrum extracted from a region on the western side of SNR 0509 (region inside the right white rectangle in Fig. 2). Seen are broad coronal lines of different ionization states of iron, calcium and sulfur (and possibly nickel) from the reverse shocked ejecta as well as broad and narrow Balmer lines from the forward shock. The gap in the spectrum at around 589 nm stems from the MUSE notch filter used to block the residual laser light from the 4LGSF system (see ‘Observations and data reduction’ section in Methods). *λ*, wavelength.

**Extended Data Fig. 2:**
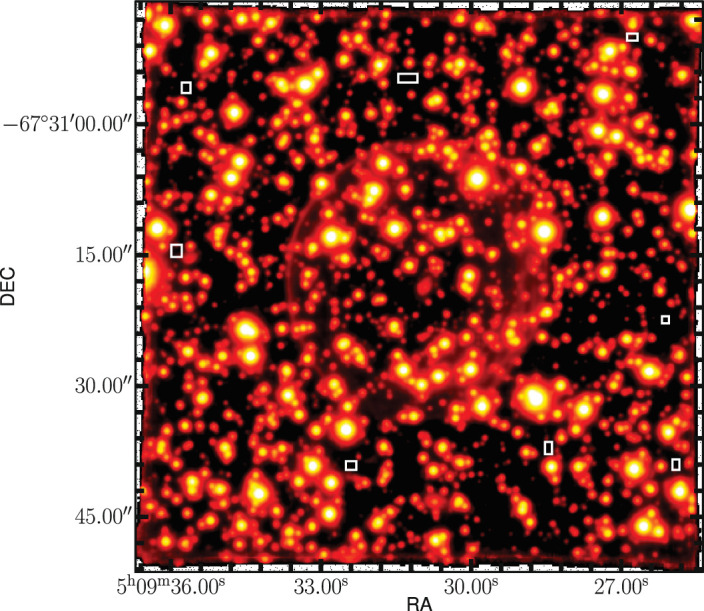
Selection of effective background. White rectangular boxes represent the area of the sky selected for additional background subtraction. This additional step greatly reduces the contamination by sky emissions.

From light echo observations, SNR 0509 is known to be part of the SN1991T-like (more luminous than average at peak brightness) sub-class of SNe Ia^41,42^. It is very young (~300–350 yr (ref. ^43^)) and located in the nearby Large Magellanic Cloud (LMC), granting us an exclusive view into the early stages of the evolution of a type Ia SNR. A few hundred years after the explosion, the inner part of the expanding ejecta is exposed by shock waves in the supernova remnant^44^ and can be spatially resolved in astronomical observations. The ejecta of SNR 0509 is expanding in a low-density ambient medium, as evidenced by the near-spherical symmetry of the forward shock. After ‘local background’ subtraction (Extended Data Fig. 2), detailed tomography and modelling of the emission of the reverse shocked ejecta in this system has been performed^44^, which reported the discovery of [Fe XIV]5303, and excesses indicating the presence of [Fe IX]8235 and [Fe XV]7060 as well as [S XII]7611. The resulting new constraints from the location of the optical emission of the reverse shocked ejecta and a set of analytical hydrodynamical supernova remnant models^44^ were used to argue that the SN1991T-like event forming this SNR should have been an energetic sub-Chandrasekhar-mass explosion^45^. Following the discovery of the optically emitting reverse shocked ejecta, our team conducted deeper optical observations of SNR 0509, which now reveal the shocked ejecta in greater detail (see Fig. 1 for a sample spectrum extracted from the western side of the remnant). In addition to the emission lines detected previously, we now also detect [Fe IX] *λ*4967, [Fe X] *λ*6375, [Fe XI] *λ*7892 and possibly [Ni XIII] *λ*4950. Importantly, we also observe broad [Ca XV] *λ*5695. The morphology of this calcium line relative to the sulfur emission reveals important clues about the nature of the supernova explosion mechanism.

**Fig. 2:**
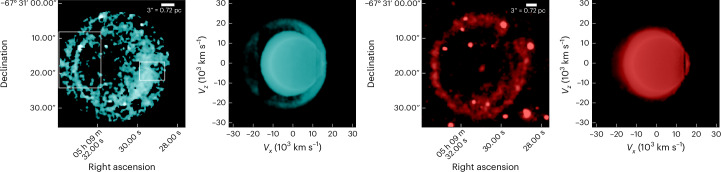
Observed and simulated shell structures in the supernova remnant 0509. Left: reverse shocked ejecta emitting in [Ca XV] in SNR 0509 obtained by integrating over a slice from 5,626 Å to 5,752 Å (for more details, see ‘Data analysis and visualization’ section). The intensity ranges from 0.05 to 0.9 × 10^−17^ erg s^−1^ cm^−2^ arcsec^−2^ Å^−1^. The area within the eastern (left) white highlighted rectangle shows the region picked for examining the double-shell structure. The region within the western (right) rectangle is the extraction aperture for the spectrum shown in Fig. 1. Left centre: integrated column (along the line of sight) of density × density × *X*(Ca), which shows a double-shell structure of calcium in the model M10_03 after 100 s of explosion (the plotted value range is 7 × 10^−20^ to 5 × 10^−13^ density^2^ *X*(*Ca*)). Right centre: reverse shocked ejecta emitting in [S XII] in SNR 0509, obtained by integrating over a spectral slice from 7,502 Å to 7,726 Å. The bright point sources in the figure are not sulfur clumps but rather stars that have strong emission lines in the wavelength range of [S XII] (for more details, see ‘Data analysis and visualization’ section). A highly red-shifted background galaxy can be observed at the same wavelength in the centre of the remnant as a diffuse red spot. The intensity ranges from 0.18 to 2.0 × 10^−17^ erg s^−1^ cm^−2^ arcsec^−2^ Å^−1^. Right: integrated column of density × densit × *X*(S), which shows a single-shell structure of sulfur in the model M10_03 (the plotted value range is 3 × 10^−19^ to 1 × 10^−13^ density^2^ *X*(Ca)). *X*(Ca) and *X*(S) represent mass fraction for the specific element. *V*_*x*_, *V*_*z*_, velocity in the *x* and *z* axis, respectively.

**Fig. 3:**
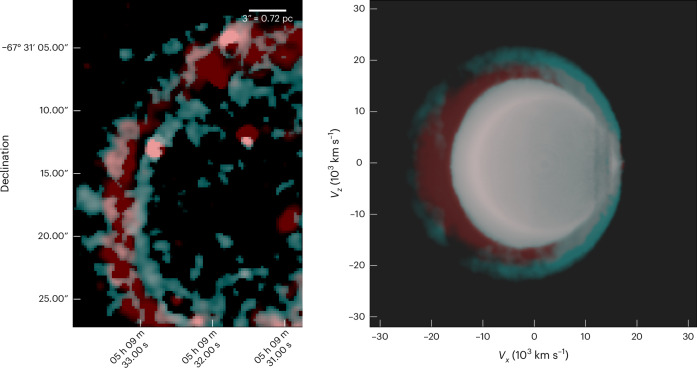
Overlaid observed ionized species showing morphological similarities with explosion simulation. Left: spatial distribution of [S XII] (red) and [Ca XV] (cyan) in the ejecta, overlaid together as observed in the eastern side of the remnant. We detect the presence of sulfur as a single shell that peaks between the two shells of [Ca XV]. Right: overlay of the integrated column of density × density × *X*(S)(red) and *X*(Ca) (cyan) from the M10_03 model, showing the presence of sulfur in between the double shells of calcium.

**Fig. 4:**
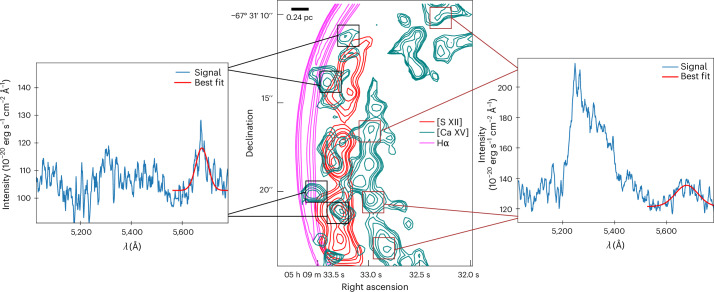
Spectral extraction and contour mapping of [Ca XV] in SNR 0509. Left: spectrum (extracted from the four black squares indicated in centre) showing the Gaussian line fit to [Ca XV] from the outer shell. Centre: surface brightness contours showing the double-shell structure of [Ca XV] (in cyan) and the positioning of [S XII] (in red) between the two calcium shells, with the forward shock (Hα in magenta) further outside, marking the outer extent of the supernova remnant. The contours correspond directly to the observations of [Ca XV] and [S XII] inside the white rectangle in the upper left panel of Fig. 2. Right: spectrum (extracted from the four maroon squares indicated in the centre) showing the Gaussian line fit to [Ca XV] from the inner shell.

**Fig. 5:**
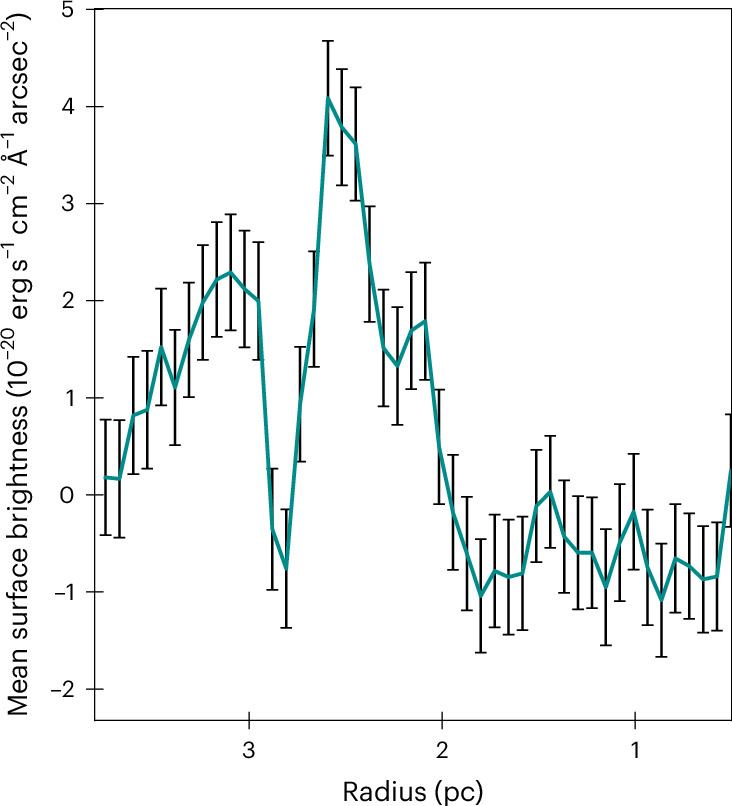
Radial profile of [Ca XV] surface brightness in SNR 0509. Mean surface brightness of [Ca XV] (integrated over 5,632–5,740 Å and binned into annuli of 1.5 spaxel), with ±1*σ* propagated standard error per pixel (as measured by the instrument), plotted against radius for the region highlighted by the white rectangle in the eastern side of the remnant (shown in the left panel of Fig. 2). The profile reveals two distinct peaks, corresponding to two shells observed in Fig. 2, separated by a region of negligible signal in between.

**Extended Data Fig. 3:**
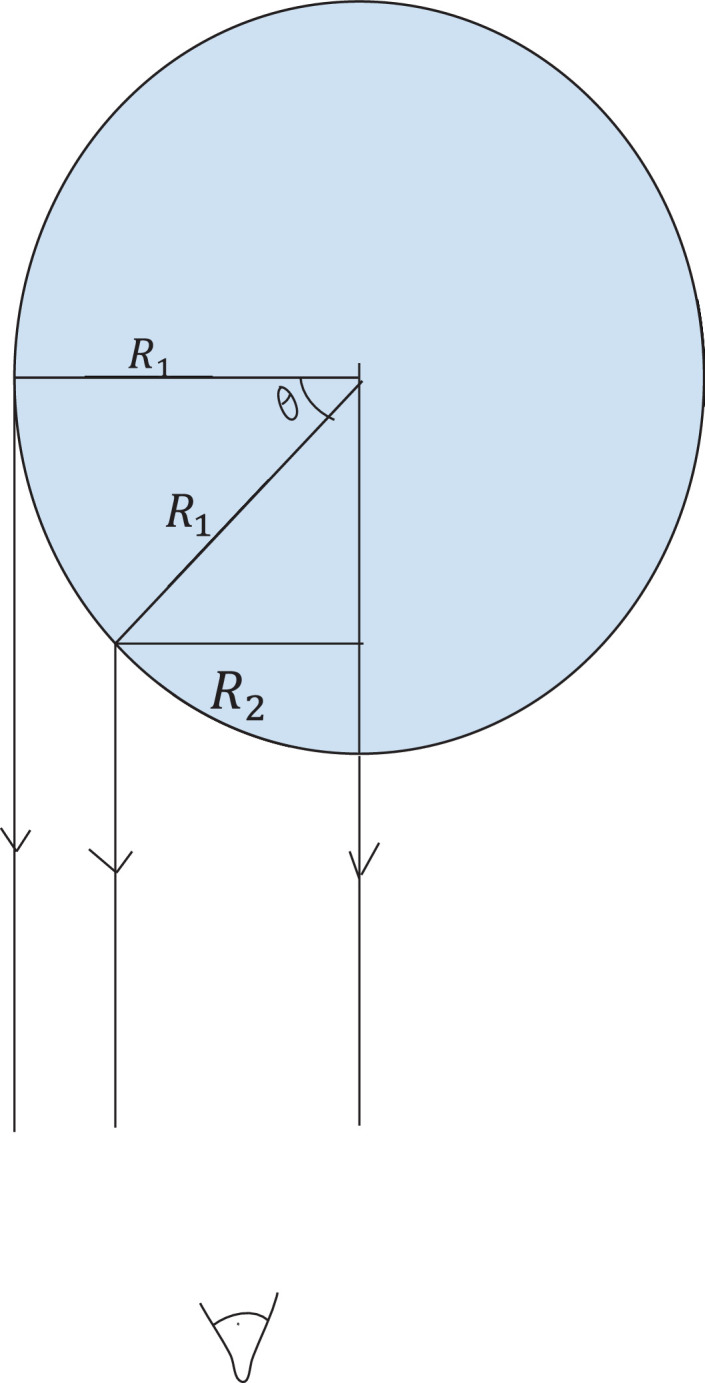
A cartoon Model of projection effects in an expanding supernova remnant. The morphology of the supernova remnant is considered, affecting the radius of an expanding spherical ejecta. This shows that if the double shell structure originated due to projection effect of two different faces of the remnant, we would expect large doppler shifts between the observed [Ca, XV] lines.

Specifically, we report here the discovery in SNR 0509 of a double-shell structure of highly ionized [Ca XV] alongside a single shell of [S XII] emission from the supernova ejecta (Fig. 2). The inward-propagating reverse shock progressively ionizes the ejecta material, exhibiting optical forbidden line emission from these highly ionized atoms of calcium and sulfur. Thus, the observed shell structures of calcium and sulfur reflect the morphological distribution of the ejecta material. The observed shell structures of these species are comparable (since the SNR is still young and expanding into a low-density ambient medium^45^) with the column density structures of the same elements in the M10_03 model^19^. M10_03 is a hydrodynamical explosion model of a double detonation with a carbon–oxygen-rich core mass of 1.028*M*_⊙_ and a He-shell mass of 0.027*M*_⊙_. The double-shell structure of ^40^Ca evident in Fig. 2 (see also Fig. 3 for overlay) is a signature of the double-detonation explosion scenario, where the outer Ca shell is formed owing to the burning of the He shell and the inner Ca shell is formed owing to burning of the carbon–oxygen core. By showing surface brightness contours, Fig. 4 illustrates the double-shell structure of calcium (cyan), with sulfur (red) peaking in between the two calcium shells, and the Balmer emission behind the forward shock that lies much further out (magenta). The position of the Balmer emission marks the shocked circumstellar medium and the observed [Ca XV] along with the [S XII] are positioned behind the forward shock. We stress again the fact that these observed emission lines of highly ionized [Ca XV] come from the ejecta that have been shocked by the (radially inward-propagating) reverse shock. The width of the Gaussian profile is proportional to the reverse shock speed. The narrow line width of the outer shell compared with the broader inner shell indicates that the reverse shock speed increases as it travels inwards, possibly owing to clumping of the ejecta. The peak of the sulfur emission as observed is spatially located between the inner and outer calcium shells, closer to the outer shell (Fig. 4, centre), which is remarkably similar to the structural morphology of the M10_03 model. We attribute the partial overlap of the sulfur and outer calcium shells to atmospheric seeing. Our spectral analysis and modelling show that, within uncertainties, the Doppler shifts of the inner and outer calcium shells are similar to one another (‘Emission line fitting’ section). This provides evidence that we are looking at two limb-brightened shells of calcium (see analysis and Extended Data Fig. 3), as predicted by the double-detonation explosion scenario. The surface brightness of the double-shell structure of [Ca XV] peaks at two radii: 1.73 ± 0.07 pc and 2.06 ± 0.07 pc from the remnant’s centre (Fig. 5). Although the observations reported here qualitatively match the signature of the double-detonation explosion model, we do not imply that the chosen model quantitatively reproduces the observations in detail. We selected this existing double-detonation model as an archetype to compare the tell-tale morphological structure of the detonations, without fine-tuning the model to achieve a best-matching fit.

The proper motion of the forward shock has been reported to be ~6,500 km s^−1^ (ref. ^46^), unsurprisingly substantially smaller than the ejecta velocity of ~25,000 km s^−1^ of the fiducial model from ref. ^19^. The simulated model only tracks the ejecta for 100 s after the explosion, whereas the observed remnant is a few centuries older. The substantial decrease in the expansion velocity is due to the remnant interacting with the circumstellar medium. This also reduces the radial extent of the ejecta and the distances between the respective shells in the supernova remnant predicted under the assumption of pure free expansion.

The spatial morphology of the observed distribution of the sulfur and calcium lines match what would be expected of a double detonation of a WD just above 1 solar mass harbouring a thin (low-mass, for example ~0.03 solar masses) helium shell. We thus conclude that SNR 0509 was the result of a double detonation initiated in a low-mass helium shell of a sub-Chandrasekhar-mass WD progenitor. This is the first direct photographic evidence of the morphological signature of a specific explosion mechanism in the remnant phase for a type Ia supernova.

## Conclusion

Our observation provides evidence from the supernova remnant phase and contributes to resolving the long-standing debate as to whether a type Ia supernova explosion is possible from a sub-Chandrasekhar-mass WD with a thin helium shell. By extension, this implies that some 1991T-like SNe are plausibly explained by double detonations of sub-Chandrasekhar mass WDs. The highest-mass explosion model from ref. ^19^ produced 0.84*M*_⊙_ of ^56^Ni, which is within the predicted range for 91T-like SNe Ia^3^. Recent observations of SN 2022joj and SN 2020eyj hint towards the possibility of a 91T-like event from the double detonation of a CO WD^4,47^. Further reports on observations of SN 2020eyj—classified as a 1991T-like event with evidence of helium-rich circumstellar material—have been speculated to be as a consequence of the double-detonation mechanism^48^. Despite the heavy limitations on three-dimensional simulation capability, and the fact that, to date, no explosion model can adequately explain 91T-like SNe, recent radiative transfer simulations that incorporate non-local thermodynamic equilibrium physics show more promise. It was recently reported that heavy elements in higher ionization states reduce absorption effects, thus bringing a wider range of He shell masses into better agreement with observed SN Ia spectra^40,49^.

While our observations prove that the double-detonation mechanism is capable of triggering an explosion in a WD star, both double-degenerate and single-degenerate origins remain possible for the evolutionary scenario^48,50^. Recent multidimensional double-detonation simulations^16,51–53^ show that, in the WD merger scenario, in addition to the primary WD undergoing a double detonation, the companion WD can also undergo a double detonation (resulting in a ‘quadruple detonation’) upon being impacted by ejecta from the exploding primary WD. Such a double double detonation could possibly also lead to the observed double-shell structure of calcium. However, self-consistent calculations of the predicted coronal line emission of the reverse shocked ejecta do not yet exist for any explosion model. While we are therefore currently unable to conclusively differentiate between the different variants of double detonations, we can say that some form of double detonation leads to type Ia supernovae.

Our discovery marks the unique potential of supernova remnant tomography in dissecting the structure of the reverse shocked ejecta; similar methods of observation can be extended to other young type Ia supernova remnants. Observations of spatially resolved inner ejecta are not possible during the explosion itself owing to the high opacity and compactness of the material. However, after the ejecta have expanded, it is possible to attain a resolved view of the nucleosynthesis and structural distribution that arose as a consequence of the type Ia supernova explosion. Detailed forward modelling of supernova remnant evolution that calculates the ejecta ionization and excitation structure for 300–800 years after explosion holds great promise to make important advances in understanding the diverse origin of type Ia supernova progenitors.

## Methods

### Observations and data reduction

SNR 0509 was observed with the MUSE^54^ optical integral field spectrograph, which is mounted on the Unit Telescope 4 (UT4) of the European Southern Observatory (ESO) Very Large Telescope on Cerro Paranal, under ESO program ID 0104.D-0104(A) (principal investigator I.R.S.). The data were acquired in service mode with the Wide-Field Mode Adaptive Optics (WFM-AO) set-up over 25 distinct nights spread over 24 months. A total of 39 individual observations had an exposure time of ~2,700 s each (corresponding to a total exposure time of ~105,300 s = 29 h 15 min on-source), while a single observation (which was ignored in our analysis) had an exposure time of 93.92 s. In the WFM-AO mode, MUSE data span the optical wavelength range from 4,690 to 9,340Å with a resolution of *R* ≈ 3,000. This mode relies on the UT4 Adaptive Optics Facility^55^, which comprises a deformable secondary mirror^56^, the AO modules GRAAL^57^ and GALACSI^58^ (of which only the latter is relevant for MUSE operations) and the 4 Laser Guide Star Facility (4LGSF)^59^ that is responsible for the creation of artificial guide stars by means of four 22-W sodium lasers. When MUSE is observing in any of its AO modes, a notch filter centred around the lasing wavelength of 589 nm is inserted in the scientific light path to avoid the contamination of data by scattered laser light. The notch filter does not prevent the contamination of MUSE observations by Raman-scattered laser photons^60–63^ (these emission lines are cleaned up by the MUSE data reduction pipeline^64^). The dip in the spectrum presented in Fig. 1 is a direct consequence of this notch filter.

We used ESOReflex^65^ version 2.11.5, and the MUSE data reduction pipeline version 2.8.9^64^ to perform a standard data reduction of our data. The standard reduction was performed using the default settings, which removes the standard and known skylines from the data. This reduction was performed on Tycho, a large-memory Linux workstation at the University of New South Wales in Canberra specifically designed for data reduction of MUSE observations. Using the MUSE pipeline, all 39 individual MUSE pixel tables were stacked together into the final mosaic analysed and discussed in this article, which has a size of 1 arcmin × 1 arcmin, with a spaxel size of 0.2 arcsec × 0.2 arcsec.

### Data processing and sky subtraction

Standard pipeline data reduction using EsoReflex performs background sky subtraction either by using pre-calculated skylines and continuum (if dedicated sky observations are available) or by computing a sky from the fraction of the field of view specified by the parameter SkyFr_2. The latter option was used here. The residual skylines present in the final mosaic remain problematic, given the low-flux scientific signals from the shocked ejecta. We have therefore implemented an additional ‘local background’ subtraction approach to further minimize these residual skylines and help with the analysis of the faint broad signals in the spectrum, similarly to ref. ^66^. The background selection was performed locally using QFitsView^67^. Eight local background regions were selected from the white-light image, avoiding stellar or SNe ejecta contamination. These areas are usually small (~30–40 spaxels), since the field is crowded with stars, and away from the SNR 0509 centre as shown in Extended Data Fig. 2.

The combined datacube (MUSE DEEP) has been corrected for Galactic extinction along the line of sight using a customized brutifus procedure (see ref. ^68^ and https://github.com/brutifus). We use a Fitzpatrick (1999) reddening law^69^ with extinction ratio, *R*_V_ = 3.1, extinction in B-band, *A*_B_ = 0.272 and extinction in V-band, *A*_V_ = 0.206, obtained through NASA/IPAC Extragalactic Database (NED) from a re-calibration^70^ of the infra-red-based dust map^71^.

### Data analysis and visualization

The highly ionized calcium ([Ca XV]) in the reverse shocked ejecta is visualized in the left panel of Fig. 2. We have integrated the spectrum from 5,626 to 5,752 Å, where we observe the [Ca XV] signal (*λ*_0_ = 5,694.80). A continuum is subtracted by selectively choosing and integrating the spectrum ranging from 5,591 to 5,608 Å and from 5,759 to 5,802 Å to minimize stars and residual noise. Since the flux of the broad emission line of calcium is very low, comparable to the background noise, we visualize the sliced data cube of calcium using a log colour scale. To minimize distracting artificial features from bright stars, we also only visualized the region inside the forward shock (as delineated by the Hα shell), since no ejecta are present outside of the forward shock for SNR 0509^43,72^ (the signal outside the forward shock is set to zero). The [S XII] map in the centre right panel of Fig. 2 was visualized by using a similar process of integrating the spectrum from 7,502 to 7,726 Å and subtracting a continuum on both sides of the signal (7,399–7,434 Å and 7,716–7,827 Å). Although this procedure works well for subtracting the stellar continuum, the resultant [S XII] signal is still left with some residual stellar emission line exactly at the same wavelength range selected for its visualization. Owing to this, a few bright stars appear as bright blobs in [S XII].

We have also analysed the hydrodynamical explosion model M10_03^19^ to compare the structural signatures of calcium and sulfur, formed as a result of the double-detonation supernova event. Because SNR 0509 is a young remnant, the reverse shock has not yet reached the centre, and as it propagates inwards, it progressively ionizes the ejecta, starting from the outer rim, affecting the calcium and sulfur in the ejecta. We calculated the ratio of the radius of the outer shell of [Ca XV] (3.53 pc) and the inner radius of [Fe IX] (2.52 pc) to be ~1.4. The inner radius of [Fe IX] in the observed ejecta marks the inwards extent of the reverse shock, owing to the fact that the reverse shock has not yet reached the centre of the explosion. The radii were calculated by fitting circles on the outer shell of [Ca XV] and the shell of [Fe IX] in SAOIMAGE DS9^73^. This ratio was then used to find the inner radii of calcium and sulfur layers in the model. The density of the elements inside the inner radius has been masked to mimic the extent of the reverse shock from the rim towards the centre in the observation. We then plotted the line-of-sight integrated column of density × density × mass fraction of calcium and sulfur, respectively. We chose this quantity for comparison with the observations because the surface brightness of the coronal lines should scale with the collision rate, which is proportional to the electron density and species ion density. For a highly ionized medium, the electron density *n*_e_ is proportional to the total particle density, giving us the density × density × mass fraction scaling.

Figure 4 (centre) shows the Balmer emission (Hα) due to forward shock and reverse shocked ejecta in terms of surface brightness contour levels. Hα (in green) contours correspond to 0.884, 0.977, 1.135, 1.254 and 1.457 10^−15^ erg s^−1^ cm^−2^ arcsec^−2^ Å^−1^, the levels of [S XII] are 1.080, 1.255, 1.533, 1.694, 2.070, 2.404 and 2.657 10^−17^ erg s^−1^ cm^−2^ arcsec^−2^ Å^−1^ and the [Ca XV] contours are 1.974, 2.411, 2.944, 3.975, 4.854 and 6.889 10^−18^ erg s^−1^ cm^−2^ arcsec^−2^ Å^−1^. The contour levels are chosen in the image created using the integration of the surface brightness per spaxel, over the binned wavelength containing their respective signals. The contours with smaller areas represent regions of higher surface brightness in contrast to larger contour areas. The formation of several small contour regions in [S XII] and [Ca XV] marks the presence of small regions with very high surface brightness, caused by the formation of high-density blobs (owing to clumping of the ejecta) behind the reverse shock. In contrast, the forward shock (Hα) is much smoother with no evidence of clumping. The above operations were carried out by the python package Astropy^74,75^ and visualized with Matplotlib^76^.

Figure 5 shows the mean surface brightness of [Ca XV] in annular bins on the *y* axis against radius on the *x* axis. The region of SNR 0509 in the north-east exhibiting the double-shell morphology most clearly is considered for the operation. The data are binned into annuli of 1.5 spaxel width, with the centre of the annuli at right ascension 05 h 09 m 31.0 s and declination −67° 31′ 18″. We masked areas most affected by stars by considering the increase in the average flux of the spectrum above 5 × 10^−20^ erg s^−1^ cm^−2^ arcsec^−2^ Å^−1^. This approach was necessary to minimize the contamination from any star as the brutifus tool is unable to subtract stellar signals in these broad coronal lines.

#### Ionization effect on ejecta

We investigated the ionization fractions in the densest part of the SNR model for 0509–67.5 favoured in recent research^44^: 1.5 × 10^51^ erg explosion energy, 1*M*_⊙_ and 0.4 AMU cm^−3^ interstellar medium density. Using an outer envelope power law of 7 for the outer 3/7 of the ejecta by mass, the densest ejecta are found at this boundary. Here, [Fe XIV] forms without any clumping, at an ionization age *n*_et_ = 2 × 10^9^ cm^−3^ s, but is maximized in ionization fraction for about a factor of 1.5× clumping in density. [S XII] requires 1.5–3× clumping, and [Ca XV] requires 3–5× density enhancement. The fact that [S XII] forms where the ejecta are predicted to be the densest while [Ca XV] forms on either side strongly implies stratification of element abundances rather than an ionization effect. This clumping most plausibly arises as radioactive Ni–Co formed in the explosion expands and compresses the non-radioactive surroundings, being consistent with the lack of clumping required for [Fe XIV].

### Emission line fitting

We selected four regions from each of the suspected double-shell structures of the [Ca XV] from the eastern region, where the shells are more distinctly visible. The spectra from the outer shell (Fig. 4, centre, black squares) and inner shell (Fig. 4, centre, maroon squares) regions are summed independently to improve the signal-to-noise ratio. Each region is ~3 × 3 spaxel, as shown in Fig. 4 (centre). We have used the Gaussian function with a defined constant and the curve-fit function from Scipy.signal to fit the signal. The curve_fit function returns the optimum parameters after fitting and the associated covariance for the values from which the errors are calculated.

We calculated the Doppler shift for both apparent shells of [Ca XV] to ensure that they are distinct structures having different distances from the remnant centre, and not simply two different regions located on different areas of the same spherical shell but having the same physical distance from the remnant’s centre. The latter might give, in projection, a false impression of a double-shell structure. We can test for and rule out such a scenario by measuring the Doppler shift of the inner shell in comparison with the outer shell. If both arcs are situated at a similar radius (that is, they are different regions of the same spherical shell), then the inner arc should be notably Doppler shifted relative to the outer arc. On the other hand, if both are found to be expanding perpendicular to the line of sight, then we are seeing two limb-brightened separate shells with a correspondingly small Doppler shift between them (Extended Data Fig. 3).

In Fig. 5, the surface brightness of the outer and inner shells of calcium reaches a maximum at a radius of around 2.06 and 1.73 pc, respectively, from the geometric centre of the remnant. For simplicity, let us assume that the ejecta are expanding radially outwards in spherical symmetry, which is likely a good assumption given the young age of the remnant and the high degree of spherical symmetry. For the ‘projection’ case described above, where the flux peaks are due to two distinct regions on the same expanding shell, the area with smaller (projected) distance from the centre will have a (projected) radius *R*_2_, which is related to *R*_1_ by an angle *θ* (Extended Data Fig. 3). Therefore, the relation between the two radii due to such a projection effect can be simply defined as ${R}_{2}={R}_{1}\cos \theta$, where *R*_1_ > *R*_2_. Therefore, *θ* = 0.57 radians. Let us assume a conservative radial expansion speed of calcium *V* = 7,000 km s^−1^ (the maximum Doppler shift calculated from iron is ~6,000 km s^−1^, and the calcium is expected to be expanding at a higher speed than iron). The Doppler velocity of the ejecta at the observed angle would then be ${V}_{0}=V\,\sin \theta$, which is ~3,800 km s^−1^. Thus, the expected difference in Doppler velocity considering the two shells as a part of the same sphere would be much higher than what is observed. We therefore rule out the ‘projection’ scenario and conclude that the calcium peaks, seen clearly in Fig. 4, arise from two physically distinct shell structures. Thus, a similar Doppler shift represents two limb-brightened edges of [Ca XV] as predicted in the models and shown in Extended Data Fig. 1. The peak wavelengths obtained from the fitting parameters are 5,677 ± 8 Å and 5,676 ± 18 Å for the outer and inner shell, respectively, indicating that the Doppler shift varies very little in the two regions: 660 ± 430 km s^−1^ for the outer shell and 730 ± 950 km s^−1^ for the inner shell.

## Data Availability

The raw MUSE data were collected at the European Organisation for Astronomical Research in the Southern Hemisphere, Chile (ESO Programme 0104.D-0104(A)) and are freely available via the ESO archive at https://archive.eso.org/cms.html. The data for the hydrodynamical simulation of the double-detonation explosion mechanism were developed at Heidelberg Institute for Theoretical Studies (HITS) and are available upon request.

## Code availability

The codes used are available upon request from the first author.
